# CD74 interferes with the expression of fas receptor on the surface of lymphoma cells

**DOI:** 10.1186/s13046-014-0080-y

**Published:** 2014-10-10

**Authors:** Zuzana Berkova, Shu Wang, Xue Ao, Jillian F Wise, Frank K Braun, Abdol H Rezaeian, Lalit Sehgal, David M Goldenberg, Felipe Samaniego

**Affiliations:** Department of Lymphoma and Myeloma, The University of Texas MD Anderson Cancer Center, 1515 Holcombe Blvd., Houston, TX 77030 USA; Immunomedics, Inc., Morris Plains, NJ 07950 USA; Center for Molecular Medicine and Immunology, Garden State Cancer Center, Morris Plains, NJ, 07950 USA

## Abstract

**Background:**

Resistance to Fas-mediated apoptosis limits the efficacy of currently available chemotherapy regimens. We identified CD74, which is known to be overexpressed in hematological malignancies, as one of the factors interfering with Fas-mediated apoptosis.

**Methods:**

CD74 expression was suppressed in human B-lymphoma cell lines, BJAB and Raji, by either transduction with lentivirus particles or transfection with episomal vector, both encoding CD74-specific shRNAs or non-target shRNA. Effect of CD74 expression on Fas signaling was evaluated by comparing survival of mice hydrodynamically transfected with vector encoding full-length CD74 or empty vector. Sensitivity of cells with suppressed CD74 expression to FasL, edelfosine, doxorubicin, and a humanized CD74-specific antibody, milatuzumab, was evaluated by flow cytometry and compared to control cells. Fas signaling in response to FasL stimulation and the expression of Fas signaling components were evaluated by Western blot. Surface expression of Fas was detected by flow cytometry.

**Results:**

We determined that cells with suppressed CD74 are more sensitive to FasL-induced apoptosis and Fas signaling-dependent chemotherapies, edelfosine and doxorubicin, than control CD74-expressing cells. On the other hand, expression of full-length CD74 in livers protected the mice from a lethal challenge with agonistic anti-Fas antibody Jo2. A detailed analysis of Fas signaling in cells lacking CD74 and control cells revealed increased cleavage/activation of pro-caspase-8 and corresponding enhancement of caspase-3 activation in the absence of CD74, suggesting that CD74 affects the immediate early steps in Fas signaling at the plasma membrane. Cells with suppressed CD74 expression showed increased staining of Fas receptor on their surface. Pre-treatment with milatuzumab sensitized BJAB cells to Fas-mediated apoptosis.

**Conclusion:**

We anticipate that specific targeting of the CD74 on the cell surface will sensitize CD74-expressing cancer cells to Fas-mediated apoptosis, and thus will increase effectiveness of chemotherapy regimens for hematological malignancies.

## Background

CD74, better known as an invariant chain (Ii) of the major histocompatibility complex II (MHC II) [1–3], is indispensable for the proper development of B cells. CD74 is internalized into the endocytic compartment, where intramembrane cleavage releases the intracellular cytosolic domain (CD74-ICD). CD74-ICD then enters the nucleus, activates NF-kB p65/RelA, and controls the differentiation of B cells through the TAFII105 coactivator [4,5]. CD74-ICD also induces expression of TAp63, which subsequently elevates expression of Bcl-2 and promotes survival of B cells [6]. Macrophage migration inhibitory factor (MIF) is the assigned ligand for CD74 and its binding activates the extracellular signal-regulated kinase-1/2 (ERK 1/2) MAP kinase (MAPK) cascade and cell proliferation [7], as well as NF-κB, through which it enhances the expression of Bcl-2 [6].

CD74 expression is rather limited in normal human tissues, but it was found to be overexpressed in more than 85% of non-Hodgkin lymphoma (NHL), chronic lymphocytic leukemia (CLL) and a majority of multiple myeloma (MM) cells [8–12]. B cells from CLL patients have higher cell surface levels of CD74 than do normal B cells, and it was shown that the activation of CD74 by MIF in CLL cells activates NF-κB and induces secretion of IL-8, which promotes cell survival and tumor progression [11,13]. CD74-mediated proliferative and pro-survival signaling can initiate or contribute to pro-carcinogenic events and enhance the survival of cancer cells.

The Fas receptor is widely expressed by many tissues, yet the extent of Fas-mediated apoptosis does not correlate with the extent of Fas expression. Hematological cancer cells are commonly resistant to Fas ligand (FasL)-induced apoptosis despite normal expression of the Fas receptor [14]. This resistance is usually not caused by Fas/FasL mutations or overexpression of apoptosis inhibitors, such as cFLIP (cellular FLICE/caspase-8-inhibitory protein) [15]. Identification of potential inhibitors of Fas-mediated apoptotic signaling in cancers and understanding of the mechanism involved are important steps necessary for the design and implementation of new targeted therapies. Reversing Fas resistance has become a primary interest in order to improve the efficacy of treatments for chemotherapy-resistant hematological cancers [16–19]. Several current treatments, like interferon gamma (IFN-γ), CD40L, and rituximab, are believed to improve responses to chemotherapy primarily through restoration of Fas apoptotic signaling [16,17,20,21]. Our goal was to identify and to target inhibitors of the Fas receptor in order to reinstate Fas-mediated apoptotic signaling in cancer cells with limited off-target effects on normal cells.

In the light of well documented CD74-mediated pro-survival effects, we aimed to examine the effect of CD74 on Fas-mediated apoptosis, which is required for effective killing of cancer cells by most chemotherapies and radiation [22–26].

## Methods

### Cell lines and drug treatments

BJAB, Raji, Ramos, Daudi, and Jurkat cells were purchased from ATCC and grown in RPMI medium with 10% FBS (HyClone) in a 5% CO_2_ atmosphere at 37°C, and split 2–3 times per week. Cell lines were authenticated by STR analysis (MD Anderson Cancer Center Characterized Cell Line Core) and regularly tested for mycoplasma (Lonza).

For drug treatment, 0.5e6 cells/mL in RPMI + 5% FBS were seeded into 24-well plates and treated with indicated doses of FasL (Enzo) or edelfosine (Sigma-Aldrich) for 20 hours, doxorubicin (Sigma-Aldrich) for 48 h, or 10 ng/mL of super FasL (sFasL; Enzo) for 16 h.

BJAB cells (1e6 cells/mL) were incubated with 50 μg/mL of milatuzumab (humanized anti-CD74 antibody, hLL1; Immunomedics, Inc.) for 10 min prior to addition of 20 μg/mL of goat anti-mouse or goat anti-human IgG (both from Jackson ImmunoResearch). Cells were washed after 30 min and subsequently incubated with either 50 ng/mL of anti-Fas antibody CH-11 (Millipore) or recombinant FasL (Enzo). Cells were harvested 24 h later and analyzed for apoptosis by propidium iodide staining and flow cytometry, as described previously [27].

### Cloning and RNA interference

To knock-down CD74 expression, BJAB and Raji cells were transduced using MISSION shRNA lentiviral particles targeting CD74 or non-target (NT) controls, according to manufacturer’s protocol (Sigma-Aldrich), and kept under selection with 1.9 μg/mL of puromycin.

The shRNA sequences from MISSION shRNA lentiviral particles were inserted between BamHI and EcoRI restriction sites in the multiple cloning cassette of pSIREN-Shuttle vector (Clontech). The U6 promoter-shRNA cassettes were then recloned to KpnI and ApaI sites of the pEPI-1 vector kindly provided by Dr. A. C. Jenke (HELIOS Children's Hospital Wuppertal, Germany) [28]. The correct sequences of inserted shRNAs were confirmed by sequencing (SeqWright).

Obtained plasmids were transfected into cells using Lipofectamine 2000 transfection reagent (Invitrogen) according to the manufacturer’s recommendations. Transfected cells were sorted 48 h post transfection based on green fluorescent protein (GFP) expression, using a flow-activated cell sorter BD FACSAria II (BD Biosciences) followed by limited dilutions and selection with geneticin (1350 μg/mL; Invitrogen) for 2 weeks.

### Western Blotting (WB)

WB was performed according to standard protocols, as described previously [29,30]. Protein expression in total cell lysates was analyzed with primary antibodies recognizing Fas (B-10), CD74 (LN-2), cFLIP, caspase-8 and caspase-3 [all at 1:1,000 dilution in 5% blotting-grade blocker (BioRad) in phosphate-buffered saline (PBS) with Tween 20], followed by anti-mouse-HRP or anti-rabbit-HRP antibodies when an unconjugated primary antibody was used. Equal loading was verified by β-actin-HRP antibody (1:10,000; Sigma-Aldrich). Visualization was achieved by Supersignal West Pico chemiluminescent substrate (Thermo Scientific). Intensity of bands was compared by densitometry using ImageJ software (NIH).

### Flow cytometry

For detection of surface Fas or CD74, cells were washed with 2% FBS/PBS and blocked with 0.025 mg/mL of mouse IgG blocking reagent (Invitrogen) at 4°C for 15 min in the dark and washed once with 2% FBS/PBS. Cells were then incubated with 3 μL of PE-conjugated anti-Fas antibody UB2 or with FITC-conjugated anti-CD74 antibody M-B741 (both from BD Biosciences) in 50 μL of 2% FBS/PBS at 4°C for 20 min in the dark. After washing cells 2× with 2% FBS/PBS, flow cytometry was performed on an BD LSRFortessa flow cytometer with Diva software (BD Bioscience).

For evaluation of apoptosis and cell death, cells were collected and resuspended in 1 mL of cold 1% FBS/PBS and stained with Vybrant® Apoptosis Assay Kit #5 according to protocol provided by the manufacturer (Molecular Probes). Flow cytometry was performed on BD FACSCanto II flow cytometer with Diva software (BD Bioscience). Data were analyzed using FlowJo software (Tree Star Inc.).

### Animal experiments and immunohistochemistry

All animal experiments were performed in accordance with the guidelines of MD Anderson Cancer Center’s Institutional Animal Care and Use Committee. C57BL/6 mice (6- to 8-weeks old; from the National Cancer Institute) were hydrodynamically transfected [31] with 100 μg of plasmid (pEF4-myc or pEF4-CD74-myc [4]; kindly provided by Dr. Idit Shachar, The Weisman Institute of Science, Rehovot, Israel). Mice were challenged with agonistic anti-Fas antibody Jo2 (0.4 μg/g; BD Biosciences) 24 h post transfection and monitored for survival up to 6 h post challenge. Livers were harvested at the time of death/sacrifice, paraffin embedded, and cut to 5 μm sections.

Formalin-fixed, paraffin-embedded liver tissue sections on microscope slides were treated with an unmasking kit (1:500; Vector Laboratories). Sections were then stained with hematoxylin and eosin (H&E; both from Fisher Scientific). Immunohistochemical staining was performed using a mouse monoclonal anti-myc antibody (1:500; Sigma-Aldrich) and a rabbit polyclonal anti-cleaved caspase-3 antibody (1:500; Cell Signaling), followed by staining and developing with an ABC staining kit (Vector Laboratories), according to manufacturer’s protocol. Stained samples were viewed using a Zeiss Axioskop 2 plus microscope (Carl Zeiss) with 4× and 20× magnification objectives.

### Statistical analysis

Differences between groups were calculated using the two-tailed Student *t* test. *P*-values less than 0.05 were considered statistically significant.

## Results

### CD74 expression in lymphoma cell lines can be suppressed by shRNAs

**Figure 1:**
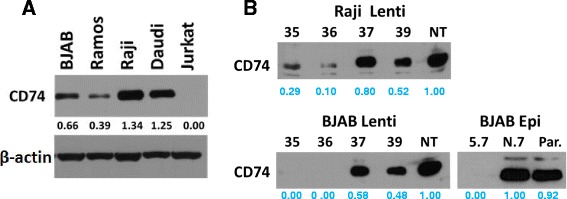
**Establishment of CD74 knock-down cells. (A)** Indicated lymphoma cell lines were harvested, lysed and CD74 expression was detected by WB using anti CD74 antibody LN-2. β-actin staining was used as a loading control; **(B)** BJAB Lenti and Raji Lenti cell lines with CD74 expression knocked-down with lentiviral vectors encoding CD74 targeting shRNAs (Sigma-Aldrich, 35, 36, 37, 39) or no-target shRNA (NT) and BJAB Epi cells with shRNAs expressed from the episomal vector (CD74 knock down cells clone 5.7, no-target cells clone N.7 and parental cells - Par) were harvested, lysed and analyzed for the expression of CD74 as in **(A)**.

To study the effects of CD74 on Fas-mediated apoptosis, we first tested CD74 expression in 4 B-lymphoma cell lines (BJAB, Ramos, Raji, and Daudi) and in the Jurkat (T-cell leukemia) cell line by WB. All four B-lymphoma cell lines expressed CD74, with the lowest expression in Ramos cells (Figure 1A). Daudi cells expressed CD74, but they are known to lack Fas on the cell surface [32]. Thus, the BJAB and Raji cell lines were selected for further analysis.

CD74 expression was first suppressed by transduction of cells with lentiviral particles encoding CD74-targeting shRNAs (#35, #36, #37, #39) or non-target control shRNA (NT). Cells transduced with particles encoding shRNA 35 and 36 showed a significant decrease of total cellular CD74 (Figure 1B) and surface CD74 (data not shown) when compared to NT shRNA controls. To exclude possible effects of a random incorporation of lentiviral sequences into the genome, we recloned CD74-targeting shRNAs 35 and NT into a mammalian-based episomal vector pEPI-1, which was shown previously to generate stable knock-downs without incorporation into the genome [28]. The generated pEPI-shRNA-35 and -NT plasmids were used to generate stable CD74 knock-downs in BJAB cells, BJAB Epi 5.7 and N.7 (Figure 1B).

### Downregulation of CD74 sensitizes cells to Fas-mediated apoptosis and to Fas-dependent chemotherapeutic agents

The pro-survival and anti-apoptotic effects of CD74 are well documented. We thus explored the effects of CD74 downregulation on Fas-mediated apoptosis that plays an important role in responses to chemotherapy.

**Figure 2:**
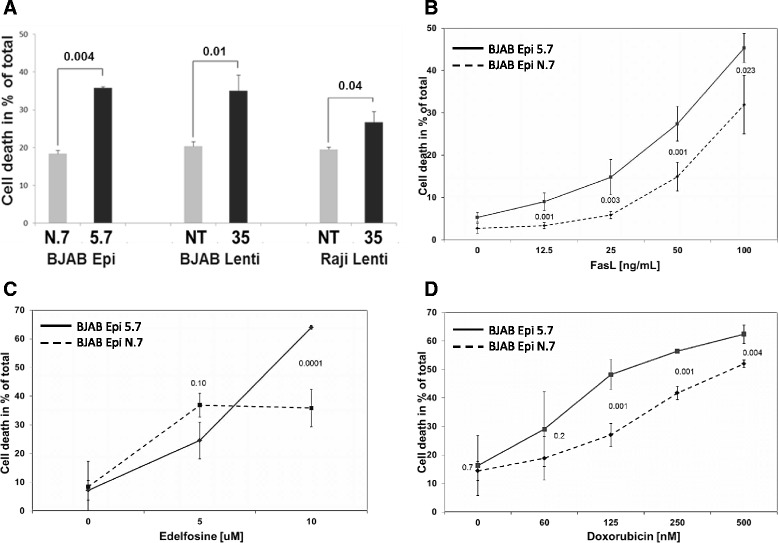
**Suppression of CD74 expression sensitizes lymphoma cell lines to Fas-mediated apoptosis and Fas-dependent chemotherapies.** Cell death was evaluated by flow cytometry in **(A)** BJAB Epi (clones 5.7 and N.7), BJAB Lenti (clones 35, NT), Raji Lenti (clones 35, NT) cells treated with 20 ng/mL of superFasL for 20 hours, **(B)** BJAB Epi clones 5.7 (solid line) and N.7 (dashed line) treated with indicated concentration of FasL for 20 hours, **(C)** BJAB Epi clones 5.7 (solid line) and N.7 (dashed line) treated with indicated concentration of edelfosine for 20 hours; **(D)** BJAB Epi clones 5.7 (solid line) and N.7 (dashed line) were treated with indicated concentrations of doxorubicin for 48 hours. Values shown represent the average and standard deviation from 3 wells. Presented results are a representative of at least two independent experiments.

Cell lines were also tested for their sensitivity to superFasL. All 3 pairs of CD74 knock-down and control (non-target) cells showed significantly increased sensitivity of CD74 knock-down cells to Fas-mediated apoptosis (Figure 2A) independently of the method used to generate the knock-downs (episome vs. lentivirus). Importantly, there were no significant differences in FasL-induced apoptosis between pairs of BJAB Epi (N.7 vs 5.7) and BJAB Lenti (NT vs. 35) cells (18 ± 0.1% vs. 36 ± 3.3% and 20 ± 1.5% vs. 35 ± 0.1%, respectively; both pairs *P* < 0.01). Because downregulation of CD74 using episomal vector (BJAB Epi 5.7) turned out to be more stable than lentiviral particles (BJAB Lenti 35 and Raji Lenti 35; data not shown), BJAB Epi cells were selected for all subsequent experiments.

Incubation of BJAB Epi cells with increasing doses of FasL revealed that cells with downregulated CD74 were more sensitive to Fas-mediated apoptosis than control (non-target) cells at all tested FasL concentrations (Figure 2B), without significant differences in basal apoptosis (0 ng/mL of FasL).

Edelfosine was shown to kill cancer cells by a ligand-independent crosslinking of Fas receptor [33]. We thus tested the effect of CD74 downregulation on edelfosine-induced cell killing. As expected, cells lacking CD74 were sensitized to the standard (10 μM) dose of edelfosine when compared to control (non-target) cells (64 ± 1% vs. 36 ± 7%; *P* < 0.001) (Figure 2C).

Doxorubicin (DOX) is a very potent anti-cancer drug used to treat lymphoma and other cancers. It is well known that cells with defective Fas signaling are resistant to killing by DOX [23]. Responses to DOX were enhanced in cells lacking CD74 (Figure 2D). Notably, cell death induced by 125 nM DOX in CD74 knock-down cells was comparable to cell death induced by 500 nM DOX in control (no-target) cells (48.2 ± 5.3% vs. 52.00 ± 1.3%; *P* = 0.8).

### Overexpression of CD74 confers resistance to Fas-mediated apoptosis

The expression of Fas receptor in the liver tissue is very high, which makes liver extremely sensitive to Fas-mediated apoptosis and injection of Fas agonists into mice causes death due to hepatic failure [34].

**Figure 3:**
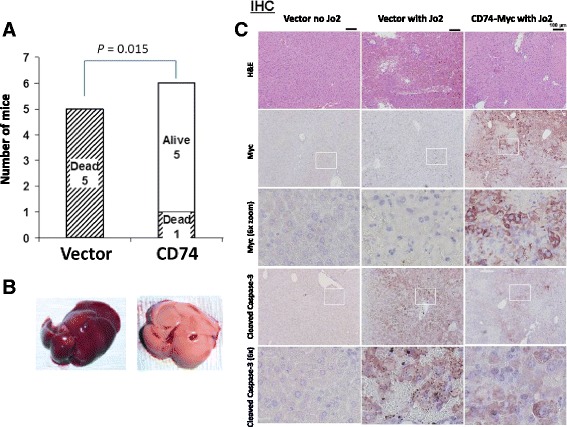
**Over-expression of CD74 protects mice from a lethal dose of agonistic Fas antibody.** Mice were transfected with an empty plasmid (vector) or plasmid encoding Myc-tagged CD74 using a hydrodynamic transfection method. Twenty-four hours post transfection, mice were challenged with a lethal dose of agonistic anti-Fas antibody Jo2 and monitored for survival up to 6 hours. **(A)** Survival of challenged mice at 6 hours; **(B)** gross appearance of livers from Jo2 challenged mice; **(C)** immunohistochemistry of liver tissues from control (vector) and Jo2-challenged (vector with Jo2 and CD74-Myc with Jo2) mice. H&E – hematoxylin and eosin staining, MYC – Myc-tag staining, Cleaved Caspase 3 – apoptosis staining.

We used hydrodynamic transfection to transiently express CD74 in mouse livers [31]. Subsequent challenge with a lethal dose of anti-Fas antibody Jo2 killed all 5 Myc-tag vector- transfected mice, while 5 of 6 mice transfected with plasmid encoding Myc-tagged CD74 (CD74-Myc) remained alive at the end of the experiment, 6 h post challenge (Figure 3A; *P* = 0.015). Livers of vector-transfected mice challenged with Jo2 appeared dark and swollen due to intensive diffuse hemorrhaging, whereas livers of mice expressing CD74-Myc showed only very little or no hemorrhaging (Figure 3B).

Hematoxylin and eosin (H&E) staining of the harvested liver tissues confirmed extensive vs. limited hemorrhaging in vector- vs. CD74-Myc-expressing livers challenged with Jo2 (Figure 3C; H&E). Expression of CD74-Myc in transfected livers was confirmed by immunohistochemistry using anti-Myc antibody (Figure 3C; Myc). Further magnification confirmed positive staining in polygon-shaped cells that contained centrally located nuclei, characteristics of hepatocytes [Figure 3C; Myc(6× zoom)].

Staining of liver sections with anti-cleaved caspase-3 antibody to evaluate apoptosis revealed higher staining intensity in the livers of vector-transfected mice compared to CD74-Myc-transfected livers (Figure 3C; Cleaved Caspase-3), confirming that expression of CD74 interferes with Fas-mediated apoptosis.

### Downregulation of CD74 enhances processing of pro-caspase-8

**Figure 4:**
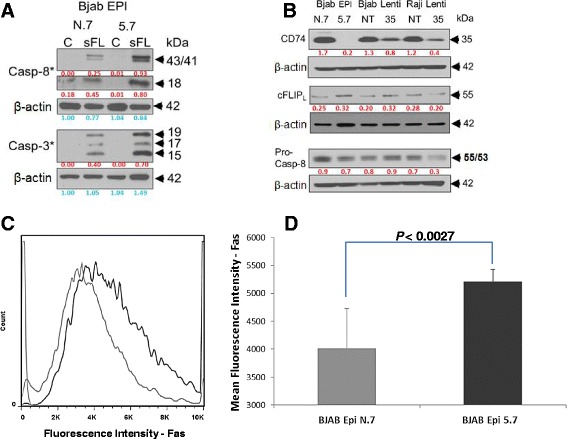
**Knock-down of CD74 sensitizes lymphoma cell lines to Fas-mediated apoptosis and increases Fas receptor levels on the cell surface. (A)** BJAB Epi cells (clones 5.7 and N.7) were treated with 20 ng/mL of super FasL (sFL) or buffer (C) for 20 hours. Cells were harvested, lysed and processing of caspase-8 and caspase-3 was detected by Western blot using antibodies specific for cleaved forms of either caspase. β-actin staining was used as a loading control; **(B)** BJAB Lenti and Raji Lenti cell lines (clones 35, NT) and BJAB Epi cells (clones 5.7 and N.7) were harvested, lysed and analyzed for the expression of cFLIP_L_ and pro-caspase-8 using specific antibodies. β-actin staining was used as a loading control; **(C)** BJAB Epi cells (clones 5.7 and N.7) were stained with PE-conjugated anti-Fas antibody UB2 and analyzed by flow cytometry. Data shown are representative of 3 independent experiments; **(D)** graphic representation of flow cytometry analysis of BJAB Epi cells (clones 5.7 and N.7) stained with PE-conjugated anti-Fas antibody UB2 and positively selected for green fluorescent protein signal. Values shown represent means and standard deviations from 3 wells.

To pinpoint which step in the Fas-mediated apoptosis cascade is affected by CD74, we treated CD74-deficient BJAB Epi 5.7 and control BJAB Epi N.7 cells with sFasL, and analyzed the processing/activation of the initiator caspase-8 and the executioner caspase-3 by WB (Figure 4A). This analysis revealed that the activation of caspase-8 and corresponding activation of downstream caspase-3 were enhanced in cells lacking CD74. This result suggests that CD74 interferes with an early membrane proximal step in the Fas signaling cascade that affects activation of caspase-8.

The membrane proximal signaling of Fas receptor can be affected by the levels of Fas receptor expressed on the cell surface and/or the efficiency of ligand binding, as well as by the inhibitor of caspase-8, cFLIP. We first focused our attention to the levels of cFLIP, expression, which is known to be upregulated by NF-κB that was shown to be activated by CD74 [6]. Comparison of protein levels in whole cell lysates from CD74 knock-down and control (non-target) cells by WB analysis showed that CD74 downregulation did not significantly affect steady-state levels of cFLIP_L_ in BJAB Epi or Lenti cells when compared to control cells (Figure 4B). The levels of cFLIP_L_ target, caspase-8, were also not affected by the CD74 knock-down (Figure 4B). This result suggested that increased sensitivity to Fas-mediated apoptosis in CD74 downregulated cells is not due to decreased expression of cFLIP_L_ or increased expression of caspase-8.

We next compared the levels of Fas receptor expressed on the surface of the cells by flow cytometry, which revealed significantly elevated levels of surface Fas in CD74 dowregulated BJAB Epi cells compared to control (non-target) cells (Figure 4C-D). Similar results were obtained in BJAB Lenti and Raji Lenti cells, which showed 17.1 ± 5.5% and 14.4% increases in the MFI in CD74 knock-down cells, respectively.

### Pre-treatment of cells with crosslinked anti-CD74 antibody sensitizes cells to Fas-mediated apoptosis

**Figure 5:**
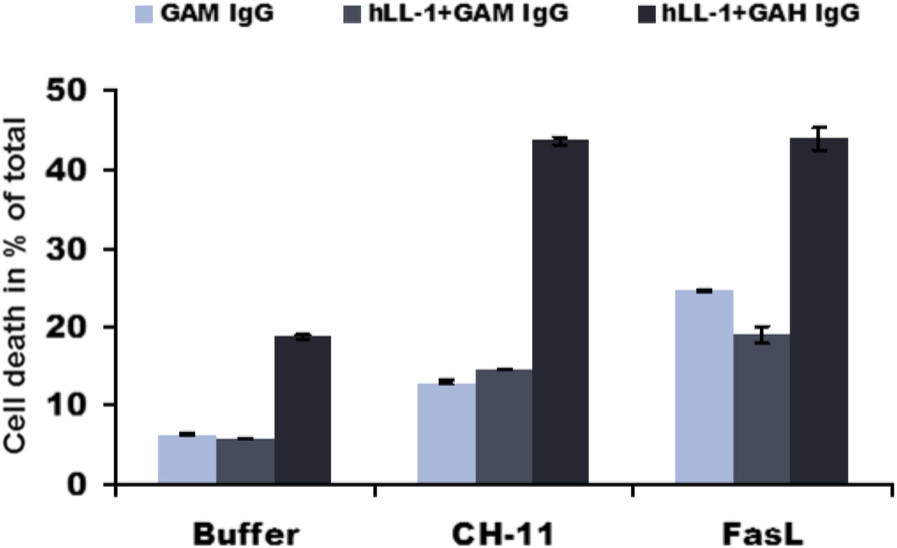
**Pre-treatment with crosslinked anti-CD74 antibody sensitizes BJAB cells to Fas-mediated apoptosis.** BJAB cells were treated with anti-CD74 antibody hLL1 crosslinked with goat anti-mouse (GAM) or anti-human (GAH) IgG followed by 50 ng/ml of either agonistic anti-Fas antibody CH-11 or FasL. Apoptosis was analyzed by flow cytometry after 24 hours. Presented values represent means and standard deviations from 3 different wells.

We next tested the ability of the humanized anti-CD74 antibody (hLL1; milatuzumab) to sensitize BJAB cells to Fas-mediated apoptosis. Pre-treatment of cells with hLL1 combined with crosslinking antibody potentiated apoptotic responses to agonistic Fas antibody CH-11, as well as FasL (Figure 5).

## Discussion

In the presented study we evaluated the effect of CD74 on the Fas-mediated apoptotic signaling in lymphoma cells by knocking down the expression of CD74 in BJAB and Raji human NHL cells. We successfully generated the first cell lines with stable knock-down of CD74 expression by lentiviral and episomal vectors expressing CD74-targeting shRNA (Figure 1B). Using these cell lines, we showed that removal of CD74 sensitizes cells to Fas-mediated apoptosis (Figure 2A-B) and subsequently also to Fas-dependent chemotherapies, doxorubicin and edelfosine (Figure 2C-D). On the other hand, the overexpression of CD74 in mouse livers protected mice from the lethality of agonistic Fas antibody Jo2 (Figure 3A) by interfering with apoptotic signaling (Figure 3C). Increased sensitivity to Fas-mediated apoptosis in cells lacking CD74 was due to increased activation/cleavage of the initiator caspase-8 and correspondingly increased activation of effector caspase-3 (Figure 4A). These results suggested that the enhancement of Fas-mediated apoptosis occurs at an immediate early step of Fas signaling at the plasma membrane – the activation of death-inducing signaling complex (DISC). MIF signaling through CD74 mediates activation of NF-κB, which is known to regulate expression of cFLIP, a well-known inhibitor of a DISC component caspase-8. However, the WB analysis of the steady-state levels of cFLIP and caspase-8 in control and CD74 knock-down cells did not reveal significant alterations of either cFLIP or pro-caspase-8 levels (Figure 4B), suggesting that the Fas signaling component responsible for the sensitization event due to CD74 removal being upstream of DISC assembly. We next tested and confirmed that removal of CD74 significantly increased the levels of Fas receptor at the cell surface (Figure 4C-D) and thus the amount of the Fas receptor available for activation. Moreover, pre-treatment of cells with crosslinked anti-CD74 antibody sensitized cells to Fas-mediated apoptosis (Figure 5).

The inherent or acquired chemoresistance is a common hindrance to the successful treatment of cancer. Multiple chemotherapies, including doxorubicin, have been shown to upregulate Fas and/or FasL in order to achieve their full effectiveness [24,25,35–39]. However, cancer cells frequently downregulate the key players in the Fas signaling cascade or upregulate apoptosis inhibitors (cFLIP, IAPs, and Bcl-2 family members) to avoid apoptosis [30,40,41]. Recently, a new category of Fas inhibitors has been recognized; cell surface receptors HGFR/c-MET, human herpesvirus-8 K1, CD44v6/v9, and nucleolin can bind and block the initiation of Fas signaling at the plasma membrane [27,29,42–44].

The increasing body of evidence points to CD74, known as the invariant chain of the major histocompatibility complex II (MHC II), functioning apart from that at MHC II. It was shown that CD74 serves as a receptor for the macrophage migration inhibitory factor, MIF. MIF-binding to CD74 activates the extracellular signal-regulated kinase-1/2 MAP kinase cascade and cell proliferation [7]. CD74 is overexpressed in a wide variety of cancers (gastric, thymic, colorectal, breast, renal and lung carcinomas, invasive thymomas, bladder, prostate and pancreatic cancers) [9,12,45–50]. Additionally, the vast majority of NHL, CLL, and MM express CD74 while limited expression is observed in normal hematopoietic tissues [8–12]. In CLL cells, MIF was shown to activate NF-κB signaling, promoting the expression of IL-8 and consequently cell survival [11,13]. The surface expression levels of CD74 in hematological cancer cells and some solid tumors correlate with poor prognosis [45,51,52].

A rapid internalization of CD74 (10^6^ to 10^7^ molecules/cell/day) in a wide range of cancer cells [53], which is not affected by the binding of the mouse monoclonal anti-CD74 antibody LL1 [54], give rise to several therapeutic approaches. LL1 linked with different radioisotopes greatly delayed growth of disseminated Raji lymphoma cells in a xenograft model and cleared tumors in 50% of the mice [55]. In the second approach, doxorubicin (DOX) conjugated to LL1 (LL1-DOX) cleared disseminated Raji lymphoma xenografts in 90% of mice. More importantly, the DOX dose used was 2.5% of the maximum tolerated dose of free DOX [56]. Multiple myeloma is in general difficult to treat. A single dose of LL1-DOX (IMMU-110, Immunomedics, Inc.) cleared multiple myeloma xenografts in 70% of mice and significantly prolonged survival [57].

The humanized form of LL1 (hLL1 or milatuzumab, Immunomedics, Inc.) alone showed promising antitumor effects in disseminated Raji and Daudi NHL xenograft models [58], and in multiple myeloma xenograft models [59]. Unlike the cell killing mechanism employed by rituximab, milatuzumab-mediated elimination of tumor cells does not involve antibody-dependent cell-mediated cytotoxicity (ADCC) or a complement-dependent cytotoxicity (CDC) [58]. It was demonstrated that milatuzumab combined with a crosslinking antibody induces aggregation of CD74 on the cell surface of CLL cells [60]. In another report, crosslinked milatuzumab induced significant apoptosis at 48 h, accompanied with activation of caspase-8 and caspase-3 [58], suggesting the involvement of an extrinsic (receptor-mediated) apoptotic pathway. Our results suggest an explanation of those results. A rapidly internalized CD74 (turnover completed in 10 minutes) can decrease the levels of surface Fas (Figure 4C-D) through its internalization from the cell surface. Crosslinked milatuzumab-induced aggregation of CD74 prevents internalization of CD74 [60] and indirectly also internalization of Fas, consequently increasing surface Fas levels and sensitizing cells to Fas-mediated apoptosis (Figure 5).

## Conclusions

We conclude that CD74 regulates Fas death receptor signaling in lymphomas by decreasing the levels of Fas receptor on the cell surface. The exact mechanism of CD74-mediated down-regulation of surface Fas remains unclear and deserves further investigation. Taking into account the frequent CD74 expression in cancers together with the impaired Fas signaling associated with chemoresistance, we extrapolate that CD74 also contributes to chemoresistance. Thus, addition of CD74-targeting antibody milatuzumab to current chemotherapy regimens can offer new therapeutic interventions, as also suggested in prior studies in multiple myeloma models [59]. Future clinical studies should determine whether milatuzumab can indeed restore Fas-signaling and chemotherapy responses in lymphoma, CLL, and potentially other CD74-expressing malignancies.
